# p73 as a Pharmaceutical Target for Cancer Therapy

**DOI:** 10.2174/138161211795222667

**Published:** 2011-02

**Authors:** Andrea Bisso, Licio Collavin, Giannino Del Sal

**Affiliations:** 1 Laboratorio Nazionale CIB, Area Science Park, Padriciano 99, 34149 Trieste, Italy; 2 Dipartimento di Scienze della Vita, Università di Trieste, Via L. Giorgieri 1, 34100, Trieste, Italy

**Keywords:** p73 tumor suppressor, TA-p73, ∆N-p73, mutant p53, apoptosis, cancer, small molecules, peptides, aptamers, cell death.

## Abstract

About half of all human tumors contain an inactivating mutation of p53, while in the remaining tumors, the p53 pathway is frequently abrogated by alterations of other components of its signaling pathway. In humans, the p53 tumor suppressor is part of a small gene family that includes two other members, p73 and p63, structurally and functionally related to p53. Accumulating evidences indicate that all p53-family proteins function as molecular hubs of a highly interconnected signaling network that coordinates cell proliferation, differentiation and death in response to physiological inputs and oncogenic stress. Therefore, not only the p53-pathway but the entire “p53-family pathway” is a primary target for cancer drug development. In particular, the p53-related protein p73 has a crucial role in determining cellular responses to chemotherapy, and can vicariate p53 functions in triggering cell death after DNA damage in multiple experimental models. The biology and regulation of p73 is complex, since the TP73 gene incorporates both tumor-suppressive and proto-oncogenic functions. However, the p73 gene is rarely mutated in tumors, so appropriate pharmacological manipulation of the p73 pathway is a very promising approach for cancer therapy. Here we provide an overview of the principal mechanism of p73 regulation, and describe several examples of pharmacological tools that can induce p73 accumulation and function by acting on upstream p73 modulators or displacing inhibitory p73 interactors. A better understanding of how the p73 pathway works is mandatory to discover additional players intervening in this pathway and has important implications for the improvement of cancer treatment with the development of new molecules or with the reposition of currently available drugs.

## INTRODUCTION

In the past 30 years, an impressive amount of clinical and basic research has focused on the p53 tumor suppressor protein, mainly because its inactivation occurs in more than 50% of all cancers [1]. Evidences demonstrate that p53 functions as a central node in a signaling pathway that prevents cancer onset and development, by sensing a variety of potentially oncogenic cytotoxic and genotoxic stress signals, and translating them into tumor-suppressive cellular responses such as cell-cycle arrest and apoptosis [2-5].

In mammals, p53 belongs to a small gene family that includes two additional paralogs, p73 and p63. They are structurally and functionally similar to p53, and have important functions in embryonic development and differentiation [6-8]. Importantly, p73 and p63 are also involved in tumor suppression, so the entire p53 family can be regarded as a signaling “network” controlling cell proliferation, differentiation and death [9]. In fact, many of the signaling pathways that convey stress signals on p53 are also active on the other family members and p73 and p63 can induce cell cycle arrest and apoptosis in response to DNA damage and other conditions that activate p53. It is conceivable that a degree of functional redundancy in the p53-family network provides robustness to the pathway, to guarantee efficient responses to stress under a wide spectrum of conditions. 

From a molecular point of view, p53 is a transcription factor and its activation/inactivation in response to stress depends on a complex repertoire of post-translational modifications and interactions with regulatory cofactors (reviewed in [2, 3]). Notably, many of the post-translational modifications that regulate p53 are also found in p73 and p63 and several p53 modulators and binding partners also interact and convey signals on p73 and p63 [9]. 

In this review we focus our attention on p73 (for regulation and function of p63, the reader can refer to several recent reviews [10-13]), since many evidences indicate that p73 is a major determinant of chemosensitivity in human tumors [14].

It is now established that pharmacological manipulation aimed at restoring the levels/functions of the p53 protein can induce tumor regression *in vivo* [15-18]. Considering that p73 is rarely mutated in cancer, pharmacological activation of the tumor-suppressive activities of p73 represents an attractive alternative strategy to treat cancer cells, in particular those where p53 is lost or mutated.

**Fig. (1):**
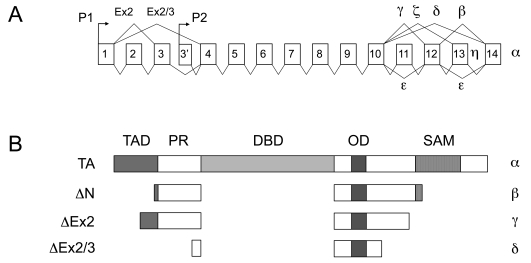
Structure of the p73 gene and encoded proteins. **A**. Structure of the p73 gene. The TP73 gene is located on chromosome 1p36.32, and is composed of 14 principal exons. Primary transcripts generated from two alternative promoters (P1 and P2) undergo differential splicing to generate multiple isoforms. **B**. Overview of the principal proteins encoded by the TP73 gene. Similar to all p53-family members, p73 contains a N-terminal transactivation domain (TAD), a proline-rich region (PR), a central DNA-binding domain (DBD) and a C-terminal oligomerization domain (OD). In p73 there is an additional C-terminal sterile-alpha motif (SAM), that is present also in p63 but not in p53. Transcripts generated from the P1 promoter encode proteins with a complete TAD, that are transcriptionally proficient; transcripts generated from the P2 promoter encode proteins that lack the TAD and are transcriptionally inactive (∆N isoforms). Additional N-terminal truncated variants are generated by splicing of the first 2 exons. Independently from promoter usage, all transcripts can undergo alternative splicing of the C-terminal exons, thus generating a combinatorial variety of isoforms (obtainable adjoining each N-terminal variant with any of the C-terminal variants). Only the full-length C-terminal variants (α isoforms) contain the SAM domain. For simplicity, only the principal C-terminal variants are represented in panel B.

The biology of p73 is complex since the p73 gene can be transcribed in a variety of different isoforms (Fig. **1**) and the resulting proteins have antagonistic properties since the TA-p73 isoforms behave as tumor-suppressors, while ΔN-p73 isoforms have the features of proto-oncogenes. Efficient pharmacological intervention on p73 must deal with this complexity. For instance, p73-activating drugs might be effective only in case of cancer cells expressing the “right” isoforms of p73 (i.e. TA-p73) and in designing new strategies it would be important to develop specific drugs, or RNA-based therapeutics, that can modulate the relative expression of ΔN-p73 and TA-p73 isoforms.

Within this conceptual framework, we provide a broad overview of the p73 pathway and its regulation, and describe current pharmacological approaches that may be targeting this pathway in cancer.

## MOLECULAR STRUCTURE OF p73

The p73 protein has a domain organization similar to p53 (and p63): full length p73 (TA-p73) contains a N-terminal transactivation domain (TAD), followed by a proline-rich sequence (PR), a central DNA-binding domain (DBD), and a C-terminal oligomerization domain (OD), involved in formation of tetramers (Fig. **1**). All p53 family proteins share a degree of sequence homology, in particular in the DBD (~70% of sequence identity) [19]. The high similarity within the DBD confers to p73 (and p63) the ability to recognize and regulate many p53 target genes (e.g. p21, PUMA, NOXA, BAX and MDM2) in several experimental models [14, 20, 21], although it remains an open question to what extent p53-related proteins regulate the same genes in physiological conditions. On the contrary, oligomerization domains are less conserved and p73 can hetero-oligomerize with p63, but not with p53 [22].

A common feature of the p53 family members is that they can be expressed in a number of different isoforms [19]. In p73, the use of an internal promoter (P2), the alternative splicing of the first exons or the use of an alternative translation start site, generate several variants with a truncated N-terminus, identified collectively as ∆N-p73 [19, 23]. ∆Np73 isoforms lack a functional transactivation domain and acquire dominant negative, anti-apoptotic and pro-proliferative functions over TA-p73 (see below). The C-terminus of the alpha isoforms contains a sterile alpha motif (SAM), and a terminal transcription inhibitory domain, not conserved in p53 [19, 20, 24]. Alternative splicing of the C-terminus gives rise to numerous additional shorter isoforms (β, γ, δ, and the less investigated ε, ζ and η), whose specific functions are still poorly characterized [7, 14, 25, 26] (Fig. **1**).

## ROLE OF p73 IN CANCER: LESSONS FROM ANIMAL MODELS

Several evidences from *in vitro* studies indicate that p73 has a tumor suppressive role. In fact, p73 can trigger cell cycle arrest, cellular senescence and apoptosis upon DNA damage, by promoting the transcription of many p53 target genes [21, 27]. Its knockdown enhances the transforming potential of p53^-/-^ mouse embryonic fibroblasts infected with the oncogenic form of Ras (Ras^V12^) [28]. Moreover, the P1 promoter, which drives the expression of TA-p73 isoforms, was found hypermethylated in primary lymphoblastic leukemias and Burkitt’s lymphomas, resulting in reduced TA-p73 expression [29, 30]. However, due to contradictory observations, it took several years to definitely accept TA-p73 as a tumor suppressor [31]. Indeed, first of all, there are no evidences that *TP73* gene deletion is causally associated to cancer [7, 32], and only a very small percentage of human tumors (less than 1%) bear p73 mutations [33, 34]. Second, the phenotype of p73^-/-^ mice (generated by deleting the exons encoding the DBD, thus affecting all p73 isoforms) offered no evidence for p73 role in cancer; p73-null mice had neurological (hydrocephalus), pheromonal, reproductive, inflammatory and behavioral defects, but showed no increased susceptibility to spontaneous tumorigenesis, since they die 4-6 weeks after birth [23].

Despite these characteristics, crucial evidences derived from the investigation of the long-term effects of p73 heterozygous mutation in mice, alone or with p53. It was shown that both the p73^+/-^ and the p73^+/-^:p53^+/-^ mice developed a more aggressive tumor phenotype, compared to p73^+/+^ and p73^+/+^:p53^+/-^ animals [35], thus indicating that p73 plays an important role in preventing cancer progression in specific tissues. Importantly, tumors developed in p73^+/-^ mice recapitulated human cancers (e.g. mammary adenocarcinomas) in which loss or reduced expression of p73 was previously reported [36-38]. 

Finally, generation of knockout mice selectively lacking the TA-p73 (TA-p73^-/-^ mice), but retaining normal expression of ∆N-p73 isoforms, provided evidences of the key role of TA-p73 in tumor suppression [39]. Compared to p73^-/- ^(generated by deleting the exons encoding the DBD, thus affecting all p73 isoforms) [23], these mice showed less severe hyppocampal dysgenesis, but increased infertility and notably a high incidence of spontaneous tumors, in particular lung adenocarcinomas. At the molecular level, it was demonstrated that TA-p73 binds to and regulates the functions of Bub1 and BubR1, suggesting a direct involvement in the Spindle Assembly Checkpoint (SAC), crucial for preventing aneuploidy and genomic instability [40]. The differences between p73^-/-^ and TA-p73^-/-^ mice not only proved the involvement of TA-p73 in tumor suppression, but indirectly pointed out the oncogenic potential carried by ∆N-p73 isoforms, thus highlighting the importance of a proper balance between TA- and ∆N-isoforms to maintain genomic fidelity in proliferating cells (see below).

## OPPOSING FUNCTIONS FOR THE SAME GENE: ∆N-p73 ACTS AS A PROTO-ONCOGENE

Many *in vitro* and *in vivo* evidences demonstrate that the N-terminally truncated ∆N-p73 isoforms have an oncogenic role. In fact ∆N-p73 is overexpressed in several tumors, among them breast [41], ovary [42, 43], prostate cancers [44], melanoma [45], neuroblastoma [46] and hepatocellular carcinoma [47, 48] and in most cases, ∆N-p73 expression is associated with therapy failure, chemoresistance, lymph node metastasis, and vascular invasion [49]. Transformation assays from *in vitro* and *in vivo* studies confirmed that enforced expression of ∆N-p73 in fibroblasts increases their colony formation capacity [50] and cooperates with RAS, c-myc and E1A in promoting transformation and tumorigenicity [51, 52].

The first demonstration that ∆N-p73 is a bona fide oncogene came from the phenotype of transgenic mice in which ∆N-p73 (in particular ∆ex2/3-p73β) was overexpressed in the liver under the control of the albumin promoter [53]. Indeed, transgenic mice displayed increased proliferation of hepatocytes, with acinar disorganization and the appearance of pre-neoplastic nodules (adenomas) that in the 83% of cases evolved in hepatic carcinoma (HCC) [53]. Accordingly, previous reports indicated ∆N-p73 overexpression in HCC patients with reduced survival [48].

*In vitro* experiments further demonstrated that the oncogenic potential of ∆N-p73 isoforms lies in their ability to block concomitantly three of the major onco-suppressive pathways – the RB, the p53, and the TA-p73 pathways – and to promote expression of anti-apoptotic genes [41, 54-57]. Indeed, ∆N-p73 promotes the inhibitory RB hyperphosphorylation by cyclin E-Cdk2 and cyclin D-Cdk4/6 kinases, thus boosting cell cycle progression caused by E2F deregulation [53, 55, 58]. Moreover, ∆N-p73 has a dominant-negative effect on both p53 and TA-p73, by competing for binding to the same target promoters and by forming transcriptionally ineffective heterocomplexes with them through the oligomerization domain [59]. Finally, at least some C-terminal variants of ∆N-p73 can modulate the expression of several anti-apoptotic genes independently of p53, thus providing a further mechanisms by which ∆N-p73 can exert proto-oncogenic functions [56, 57, 60, 61].

Altogether these data established that the expression levels of ∆N-p73 and the ratio of ∆N to TA-p73 isoforms are crucial parameters to determine the net effect of p73 and to predict the effectiveness of chemotherapy [14, 62-64]. For this reason, factors that can perturb their relative levels are functionally relevant. For instance the overexpression of the oncogenic form of H-Ras (H-Ras^V12^) in primary fibroblasts triggers the down-regulation of TA-p73 expression and favors the increase of ∆N-p73 isoform. The unbalanced TA-/∆N-p73 ratio overcomes TA-p73 tumor suppressor functions, thereby promoting anchorage independent cell growth [65]. 

Finally, it is worth to mention that both p53 and TA-p73 bind the internal P2 promoter of *TP73*, thus promoting transcription of the ∆N-p73 isoforms [54, 66, 67]. These data imply the existence of a negative feedback loop between ∆N-p73 and p53/TA-p73 that may self-restrict their transcriptional activities.

## REGULATION OF THE p73 PATHWAY

Similar to p53, p73 is embedded in a complex regulatory pathway that allows to tightly control and finely tune its activation and functions. Due to the relevant role that p73 (both TA- and ∆N) plays in tumorigenesis, the dissection of the p73 pathway and the knowledge of the interconnections between its components, is fundamental to conceive new therapeutic approaches. We have collected here some examples proving that it is theoretically and practically possible to empower either TA-p73, or block ∆N-p73, by targeting/displacing proteins and enzymes involved in their regulation. To better contextualize these examples, it is useful to review the principal mechanisms of p73 regulation.

## MODULATION OF p73 TRANSCRIPTION

A number of studies have investigated the transcription of TA-p73 isoforms driven by the P1 promoter, while the regulation of the P2 promoter is much less clear [54, 66, 67].

One important transcription factor involved in the expression of TA-p73 isoforms is E2F1, which can induce apoptosis, at least in part, by binding to its target sequences in the P1 promoter. [68-70]. A repertoire of post-translational modifications of E2F1 modulate its activity on the P1 promoter. In fact after genotoxic stress, phosphorylation by Chk1/2 kinases [71] and acetylation by histone acetyltransferase PCAF have been shown to modify E2F1 and to trigger trascription of TA-p73 [72, 73], while E2F1 deacetylation by Sirt1 leads to inhibition of TA-p73 transcription [74]. Also methylation/demethylation of E2F1 by Set9 and LSD1 enzymes respectively have been demonstrated to be critical for E2F1-dependent regulation of TA-p73 and induction of apoptosis [75].

The activity of E2F1 on transcription of TA-p73 is also dependent on other factors that bind to the P1 promoter. For instance, the transcriptional repressor C-EBPα inhibits [76] while the Ying Yang 1 (YY1) transcriptional activator promotes p73 expression [77]. 

The transcriptional repressor δEF1/ZEB was reported to bind to a regulatory region in the first intron of p73, exerting a silencer activity on E2F1-dependent transcription of TA-p73 [78]. 

Also c-myc and the viral oncogene E1A were described to cause the up-regulation of p73 levels and to trigger p73-dependent apoptosis *in vitro*, suggesting that, similarly to p53, p73 might be involved in a tumor suppressor circuitry that responds to restrict aberrant oncogenic signals *in vivo*, as recently demonstrated in the case of c-Myc-driven lymphomagenesis [79-81]. 

## REGULATION OF p73 PROTEIN LEVELS

An important regulator of p73 stability is the NEDD4-like ubiquitin ligase Itch, that recognizes a PY motif (the PPxY aminoacid sequence) in the C-terminal region of p73 that is not present in p53. Upon interaction, Itch triggers p73 poly-ubiquitination and proteasomal degradation [82]. DNA damage causes down-regulation of Itch through a poorly understood mechanism, thus allowing the stabilization and activation of p73 [82-85]. 

The interaction between Itch and p73 can be regulated by proteins that indirectly affect p73 levels and activity. One example is provided by the transcriptional co-activator YAP1 (Yes-associated protein 1), a key node of the Hippo signaling pathway, active in controlling organ size by modulating cell polarity, proliferation, apoptosis, and stemness [86]. YAP1 competes with Itch for binding to the PY motif of p73, thus allowing its stabilization [87]. In addition, YAP1 is also a transcriptional co-factor of p73 that increases its transcriptional activity upon DNA damage [88-90].

Mouse double minute 2 (MDM2) protein is the main E3 ubiquitin ligase controlling p53 stability [91, 92]. MDM2 can also bind to p73, however this interaction does not trigger p73 degradation. Rather, MDM2 blocks p73 transcriptional activities interfering with its modification by the acetyl-transferase p300/CBP [93, 94]. Interestingly, although MDM2 does not trigger p73 ubiquitination, it can catalyze p73 neddylation (the conjugation of the small ubiquitin-like protein NEDD8 [95, 96]), which inhibits p73 transcriptional activity [97].

Other proteins involved in p73 degradation are represented by the F-box protein FBXO45, that binds to and promote the ubiquitination and degradation of both TA- and ∆N-p73 isoforms [98] and the U-box-type E3/E4 ubiquitin ligase UFD2a which interacts with the SAM domain of TA-p73α, triggering its proteasomal turnover in a ubiquitination-independent manner [99]. Finally, NF-kB activation was also reported to impair TA-p73α functions by inducing its ubiquitin-dependent proteasomal degradation [100] and also by promoting MDM expression [101].

Fewer proteins were reported to specifically promote the stabilization of p73. One is NEDL2, a NEDD4-related protein that binds and ubiquitinates p73, surprisingly increasing its stability [102]. Another is the NAD(P)H quinone oxidoreductase 1 NQO1, that binds to p73 and prevents its ubiquitin-independent degradation by the 20S proteasome [103].

Finally, some factor might regulate differentially the protein levels of TA-p73 and ∆N-p73 isoforms. One example is provided by c-Jun that, upon stress, prevents degradation of TA-p73 [104] and at the same time triggers degradation of ∆N-p73 by the non-classical polyamine-induced antizyme (Az) pathway. The Az antizyme (a small protein initially identified as an inhibitor of ODC (ornithine decarboxylase), the first key enzyme in the polyamine biosynthesis pathway [105]), whose expression is regulated by polyamines during mRNA translation, binds to and accelerates the degradation of several substrates (e.g. ODC, Smad1, cyclinD1, and Aurora-A) via the proteasome. In the case of ∆N-p73, Az expression is promoted by c-Jun upon genotoxic stress, thus leading to proteasome-mediated ubiquitin-independent degradation of ∆N-p73 [105, 106]. Recently, also the p73-Induced RING 2 protein (PIR2), a ring-finger domain ubiquitin ligase, was reported to regulate the ratio of TA- to ∆N-p73 isoforms. Notably, PIR2 is a transcriptional target of TA-p73 that preferentially degrades ∆N-p73, thus releasing TA-p73 and triggering apoptosis following DNA damage [107].

## POST-TRANSLATIONAL REGULATION OF p73: PHOSPHORYLATION AND ACETYLATION

A complex repertoire of post-translational modifications and protein-protein interactions control the levels and functions of all the members of the p53 family [9]. Besides ubiquitination and neddylation (see above), p73 is regulated by phosphorylation and acetylation on multiple residues. Most of these modifications are triggered by DNA damaging and chemotherapeutic drugs (e.g. cisplatin, doxorubicin and taxol) that stimulate accumulation and activation of p73 [64, 108].

One important post-translational modification of p73 is phosphorylation by the non-receptor tyrosine kinase c-Abl that targets multiple residues of TA-p73α. c-Abl interacts with p73 and is required for its phosphorylation upon γ-radiation or cisplatin treatment, contributing to TA-p73α induction and p73-dependent apoptosis [83-85]. Notably, c-Abl phosphorylates and stabilizes also ∆N-p73 isoforms, probably favoring their anti-apoptotic roles [109].

p73 is also controlled by kinases involved in the DNA-damage response (DDR [110]). Indeed, the serine-protein kinase ATM (Ataxia Telangiectasia Mutated) activates c-Abl [111, 112] and is required for p73 phosphorylation upon ionizing radiation [85]. Also the checkpoint kinase Chk2, a downstream effector of ATM, can phosphorylate p73, increasing its transactivation activity and pro-apoptotic functions [113]. Notably, ATM may modulate p73 levels through an additional mechanism: after cisplatin treatment ATM phosphorylates IKK-α, one of the two catalytic subunits of the I kappa B kinase (IKK) complex, that accumulates in the nucleus and promotes p73 stabilization, thus inducing p73-dependent apoptosis [114, 115].

Genotoxic stress activates other two pathways, the p38 MAPK and the JNK pathways, that mediate p73 activation. In tissue culture, JNK-mediated phosphorylation of TA-p73 increases p73 transcriptional activity and stimulates induction of apoptosis after cisplatin treatment [116]. Similarly, p38 phosphorylates threonine residues of p73 and this event is fundamental to mediate p73 activation by c-Abl [117]. These phosphorylations are critical for recruiting the prolyl isomerase Pin. This enzyme catalyzes the conformational changes of TA-p73 required to its accumulation and acetylation [118, 119] and to direct p73 to its target promoters after drug treatment [118]. These conformational changes could also modulate the interaction of p73 with elements of chromatin remodeling complexes, such as the bromodomain-containing 7 BRD7 that binds and regulates all p53 family members ([120, 121] and GDS unpublished observations).

Finally, the protein kinase C(δ (PKCδ), which is involved in DNA damage induced apoptosis, can also phosphorylate TA-p73β at serine 289, mediating its stabilization and activation [122].

## REGULATION OF p73 BY PROTEIN-PROTEIN INTERACTIONS

Besides post-translational modifications, interaction with other proteins can deeply affect p73 functions. One example is the association with the ASPP (Ankirin repeats, SH3 domain, proline-rich protein) proteins, cofactors that can bind p73 as well as p53 and p63 [123-125]. Association with ASPP proteins differentially affects p73 functions: in particular, ASPP1/2 stimulate p73 transcriptional activity, while iASPP inhibits p73 activation and p73-mediated apoptosis [125, 126].

A further important level of regulation is provided by the interaction of p73 with other two members of the p53 family, in particular the “dominant negative” ∆N-p63 isoforms and the oncogenic tumor-derived mutant forms of p53 (mut-p53). ∆N-p63 behaves similarly to ∆N-p73: it can associate with TA-p73, forming inactive hetero-oligomers, thus competing for binding sites on promoters and inhibiting TA-p73 transcriptional activity and p73-induced dependent apoptosis [127]. Apparently, this proto-oncogenic function of ∆N-p63 is particularly relevant in head and neck squamous cell carcinomas (HNSCC), where ∆N-p63 expression correlates with chemo- and radio-resistance. Accordingly, experimental knockdown of endogenous p63 in HNSCC cells by RNA interference resulted in induction of TA-p73-dependent apoptosis [127]. This inhibitory association was detected also in cell lines derived from triple negative breast cancers (TNBC, lacking estrogen and progesterone receptors and Her2 amplification), where treatment with cisplatin can activate the c-Abl/TA-p73 axis and induce apoptosis. This outcome requires the dissociation of TA-p73 from ∆N-p63, that normally promotes the survival of breast cancer cells by inhibiting TA-p73 functions [62]. It follows that ∆N-p63 levels may be crucial in determining TNBC sensitivity to cisplatin.

Finally, one of the most relevant inhibitory interactions of p73 is the formation of complexes with mutant p53. In fact, in almost half of all tumors p53 bears point mutations that impair its transcriptional activity [1]. Besides the loss of wt-p53 functions, these mutants acquire new oncogenic properties (“gain of function”, GOF) that actively sustain tumor development and progression [1]. One of the mechanism by which mut-p53 exerts its GOF is the ability to bind and inhibit p73 and p63, blocking transactivation of downstream targets involved in the induction of apoptosis and cell-cycle arrest [128-134]. An important modifier of the GOF of mut-p53 is the presence of a polymorphic site at codon 72, that encodes either an arginine (72R) or a proline (72P) [128, 135, 136]. While wt-p53 72R can induce apoptosis much better compared to the more frequent 72P variant [136], mut-p53 72R confers higher chemoresistance to cancer cells than mut-p53 72P [128, 135, 137]. Notably, mut-p53 72R isoforms interact with p73 and impair p73-dependent transcription more efficiently than 72P mutants [128]. This difference may have relevant implications for the potential p73-dependent chemotherapeutic responses of cancers bearing mut-p53.

## p73 AS A NOVEL TARGET FOR ANTICANCER THERAPIES

Substantial evidence indicates that p73 has tumor-suppressive functions and can vicariate, under some conditions, the antioncogenic functions of p53 in p53-null or mut-p53 expressing cells. Therefore, proper stimulation of TA-p73 expression and function in cancer cells is a promising objective for pharmacological intervention. In principle, considering that E2F1 triggers TA-p73 transcription and E2F1 responds to genotoxic stress [161], most conventional chemotherapeutic drugs can be expected to potentially activate the E2F1-TA-p73 axis, and thus increase the levels of TA-p73. Nonetheless, not surprisingly, TA-p73 activities are frequently dampened in tumors by mutation or deregulated expression of p73 modulators or co-factors.

Taking into account the biology of p73 in cancer cells, this pathway can be activated by three broad categories of drugs: 1) compounds that can increase TA-p73 levels, for instance by stimulating its transcription or by inhibiting degradation of the protein, 2) drugs that can modulate the expression or functions of TA-p73 upstream regulators, for instance kinases and acetylases, and 3) molecules that can displace/disentangle p73 from inhibitory interactions with other cellular proteins, most notably ΔN-p73 isoforms and mutant p53.

## ACTIVATION OF p73 BY UNCOUPLING OF NEGATIVE REGULATORS

An intensive field of research is focusing on the identification of compounds able to inhibit/displace the interaction between p73 and p53 mutants; targeting this mechanism is particularly attractive since it is a distinguishing feature of transformed cancerous cells [162]. 

### RETRA.

One such compound is the small-molecule RETRA (REactivation of Transcriptional Reporter Activity) [138]. This compound promotes the expression of a set of p53-regulated genes in mutant p53 expressing cells, thus leading to the suppression of cell growth both *in vitro* and in mouse xenografts. Importantly, this activity is dependent on expression of mut-p53 and p73: in fact, RETRA effects were ascribed to its ability to release p73 from the inhibition exerted by mut-p53 and to promote an increase in p73 levels. Considering that RETRA administration was well tolerated in mice, this molecule represents a promising compound to reactivate p73 function in tumors [138].

### PRIMA-1.

Additional compounds able to release p73 from the inhibitory binding of mut-p53, are the low molecular weight compounds PRIMA-1 (p53 Reactivation and Induction of Massive Apoptosis), and its analog PRIMA-1^MET^ (APR-246, already tested in the clinical trial NCT00900614) [141, 163]. PRIMA-1 was shown to reactivate wild-type p53 (wt-p53) functions through covalent binding to the core domain of p53 mutants, thus restoring their DNA binding activity and inducing apoptosis in tumor cells [141, 163]. The restoration of wt-p53 conformation promoted by PRIMA-1 allowed to speculate that PRIMA-1 could also release p73 from the inhibitory binding of mut-p53. In line with this hypothesis, PRIMA-1 was reported to cooperate with the chemotherapeutic drug cisplatin (that stimulates p73 functions) in inducing tumor cell apoptosis and inhibiting tumor growth in xenografts [142].

### Small Peptides.

In addition to chemical compounds, alternative approaches aimed at identifying small peptides capable to enhance p73 tumor suppressive functions by releasing p73 from the inhibitory association with mut-p53 or iASPP. For instance, Small Interfering Mutant p53 Peptides (SIMP) derived from the core domain of p73 can indeed target the mut-p53/p73 complex, thus restoring p73 transcriptional functions and enhancing the apoptotic response to doxorubicin and cisplatin in transformed cells bearing mutant p53 [140]. Similarly, we have identified small peptide aptamers [139] that specifically bind to mutant p53 and trigger apoptosis only in mut-p53 expressing cells [139]. Further studies are required to verify whether the release of TA-p73 from inhibitory association with mut-p53 contributes to their effect [139].

An approach using small peptides has also been successfully employed to release p73 from the inhibitory association with the ASPP family member iASPP. More specifically, a hybrid peptide of 37 aminoacids (“37aa peptide”) corresponding to residues 118–142 and 171–181 of human p53, works as an effective inhibitor of p73 binding by iASPP [126]. Indeed, this peptide induces apoptosis selectively in cancer cells (and not in normal cells) by interacting with iASPP and restoring p73 functions [126]. Importantly, in mice, systemic *in vivo* delivery of a transgene expressing this peptide can trigger p73-dependent tumor regression, thus confirming that it is possible to specifically activate tumor suppressor functions of TA-p73 *in vivo* by uncoupling the inhibitory action of iASPP [126].

### Nutlin-3.

As already mentioned above, one important negative regulator of p73 functions is MDM2. The small molecule Nutlin-3 is a powerful inhibitor of MDM2 that blocks its interaction with p53, thus allowing p53 stabilization and p53-dependent cell cycle arrest and apoptosis [143]. Notably, Nutlin-3 treatment can also block proliferation and induce apoptosis in cells lacking p53, or expressing mutant p53 [144, 145], suggesting that p53-independent effects of Nutlin-3 may be mediated by p73. Taking into consideration that both p53 and p73 interact with the same hydrophobic pocket of MDM2, it is possible that Nutlin-3 could prevent formation of MDM2/p73 complexes [164]. Indeed, it was reported that Nutlin-3 effectively displaces MDM2 from p73 and, unexpectedly, causes TA-p73 accumulation in p53 null cells, thus triggering TA-p73-dependent transcription of classical p53 targets (e.g. Noxa) involved in the induction of apoptosis [164]. Moreover, functional cooperation between Nutlin-3 and doxorubicin, a commonly used chemotherapeutic drug, was observed in highly chemoresistant, p53-null LA155N neuroblastoma cells [145]. Mechanistically, Nutlin-3 acts by releasing the E2F1/p73 complex from inhibitory MDM2 binding, while doxorubicin promotes phosphorylation of E2F1 by Chk1/Chk2 kinases, thus stabilizing E2F1 and promoting E2F1-mediated TA-p73 transcription and activation (see above). Altogether, these data suggest that p53-null tumors that retain the expression of TA-p73, will respond to the concomitant treatment with Nutlin-3 and DNA damaging agents [145].

### Panobinostat.

Despite increasing knowledge on the mechanisms regulating p73 stability and degradation, compounds that inhibit p73-specific ubiquitin ligases have not been identified so far. Nevertheless, modulating the levels or activities of ubiquitin-ligases would be highly relevant to control the amount of p73 in a cell. A striking example is provided by the action of a micro-RNA (miRNA), miR-106b, whose expression correlates with stabilization and activation of p73 in chronic lymphocytic leukemia (CLL) [148]. miRNAs are small non coding RNAs that finely regulate gene expression by binding the 3’UTR of their target mRNAs, thus reducing their translation, stability and/or changing their localization [165]. miR-106b interacts with the 3’UTR of Itch mRNA, and is a negative regulator of Itch protein levels. Interestingly, Panobinostat (LBH589), a pan deacetylase inhibitor tested in several clinical trials, stimulates the E2F1- and myc-mediated transcription of miR-106b. As a consequence, by indirectly modulating the translation of Itch, Panobinostat promotes the stabilization and activation of p73 that, in turn, induces PUMA expression and apoptosis in CLL cells [148].

## ALTERATION OF THE TA- p73 ΔN-p73 RATIO

As already mentioned, inhibition of TA-p73 functions is frequently observed in cancer as a consequence of ΔN-p73 overexpression (see above). For this reason, selective pharmacological modulation of ΔN-p73 levels may represent a valid approach to promote TA-p73 functions. A number of small molecules appear to be able to modulate the ration between TA- and ΔN-p73, often via poorly understood mechanisms.

### Celecoxib.

In this regard, the cyclooxygenase inhibitor Celecoxib was reported to reduce expression of ∆N-p73 isoforms in primary and immortalized neuroblastoma cell lines [166]. Apparently, low doses of Celecoxib negatively affect E2F1, at least in part through inhibition of COX2. Reduced E2F1-dependent transactivation of the p73 promoter down-regulates expression of ΔN-p73 and TA-p73α, but increased TA-p73β protein levels. The mechanism behind the unexpected effect of Celecoxib on selected p73 isoforms is still unknown. However, the increased amount of TA-p73β and the concomitant down-regulation of ΔN-p73, are sufficient to promote drug-dependent growth arrest and apoptosis in treated cells [166]. These results support the potential efficacy of combinations of cyclooxygenase inhibitors with chemotherapy in tumors, and indeed this is matter of ongoing studies (for instance clinical trial NCT00346801, aimed at testing the efficacy of combined treatment with Celecoxib and cisplatin in non-small cell lung cancer).

### Arsenic Trioxide and MEK1 Inhibitors.

The altered balance between ΔN- and TA-p73 isoforms has been shown to be important also in acute promyelocytic leukemia (APL). APL is characterized by the t(15;17)(q22;q12) translocation, that generates the oncogenic PML-RARα chimeric protein [167]. Treatment with arsenic trioxide (ATO) causes both apoptosis and differentiation of APL cells and, in combination with retinoic acid, ATO definitively cures most patients affected by this form of leukemia [167]. Interestingly, treatment with ATO in combination with two MEK1 (mitogen-activated protein kinase kinase 1) inhibitors (PD98059 and PD184352) greatly enhanced apoptosis of primary cells from APL patients, as well as immortalized APL and eritroleukemia cell lines [149, 150, 168]. This effect is p53-independent and relies mainly in the change of ΔN-/TA-p73 ratio. Indeed, while ATO treatment alone stimulates the expression of both TA- and ∆N-p73 isoforms, the co-administration of MEK1 inhibitors results – by an unknown mechanism – in preferential induction of TA-p73, thus increasing p73-dependent apoptosis both *in vivo* and *in vitro *[149, 150]. 

### ∆N-p73 Antisense Oligonucleotides.

An alternative, RNA-based strategy aimed at targeting all ΔN-p73 isoforms seems to be very promising [169]. By coupling locked nucleic acid (LNA) antisense oligonucleotide gapmers to magnetic nano-bead polyethyleneimine (MNB/PEI) carriers, antisense oligonucleotides selectively targeting ∆N-p73 transcripts could be efficiently delivered to tumor cells both *in vitro* and *in vivo*. These molecules induce the selective degradation of ∆N-p73 mRNA variants; down-regulation of aberrantly expressed ΔN-p73 isoforms in neoplastic cells as well in rapidly growing tumor xenografts resulted in inhibition of tumor growth and sensitization to chemotherapy, supporting the further development of ΔN-p73 inhibitors as potentially new anticancer agents [169].

## OTHER MOLECULES THAT DIRECTLY OR INDIRECTLY PROMOTE p73 EXPRESSION AND FUNCTIONS

In the last years, several other molecules were demonstrated to increase p73 expression and stimulate its functions, mainly by acting on the upstream signaling converging on key p73 regulators. Although their mechanism of action is often unclear, it is important to consider that most of these compounds are already in use for the therapy of cancer, or are under investigation in clinical trials.

### Forodesine.

Chronic lymphocytic leukemia often presents deletions of *TP53* and ATM that are associated with poor overall survival, shorter time to disease progression and resistance to treatment [170]. Forodesine (also known as Immucillin H) is an inhibitor of the purine nucleoside phosphorylase and is already employed in several clinical trials. Interestingly, forodesine was reported to have antileukemic activity in primary cells from CLL patients, when administered alone or in combination with other chemotherapeutic drugs, regardless of p53 and ATM status [146, 171]. Forodesine effects may be at least in part mediated by p73, since treated cells have increased levels of the pro-apoptotic proteins TA-p73 and Bim. Indeed, through an unknown mechanism, forodesine increases the transcription of TA-p73 in CLL cells, thereby promoting its accumulation and functions [146].

### Lenalidomide.

Lenalidomide (also known as CC-5013, or Revlimid) is an immune-modulatory agent that is giving promising results in several clinical trials for CLL [172, 173]. In CLL cells, lenalidomide stimulates transcription of CD154 (the CD40-ligand) via the NFATc1 (Nuclear Factor of Activated T cells c1)/NF-kB complex, and promotes stabilization of CD154 mRNA via the phosphoinositide-3 (PI3)-kinase pathway [147]. Through a poorly understood mechanism, increased levels of CD154 trigger c-Abl dependent activation of p73 expression, that, together with increased levels of pro-apoptotic BID and DR5 proteins, sensitizes p53-deficient CLL cells to apoptosis induced by ﬂudarabine, or mediated by CD95 [174].

### Enzastaurin.

As already mentioned, regulation of p73 functions by c-jun represents a further mechanism to control p73 activity in cancer cells. In this regard, an interesting molecule is Enzastaurin (LY317615.HCL), a small inhibitor of protein kinase C (PKC) that gave promising results in preclinical studies in several tumor cells [175]. More specifically, studies in multiple myeloma (MM) cells revealed that Enzastaurin promotes the accumulation of β-catenin by blocking phosphorylations that are required for its proteasomal degradation. Nuclear β-catenin stimulates c-Jun-dependent induction of p73 (see above) that, in turn, triggers apoptosis [153]. Enzastaurin is currently being tested in a clinical trial for multiple myeloma (NCT00718419).

### Thymoquinone.

Another compound proposed to activate the p73 pathway is the anti-neoplastic drug thymoquinone (TQ), a potent cytotoxic and genotoxic drug effective in several cancer cells [176]. TQ has been reported to generate intracellular ROS, thereby inducing DNA damage and apoptosis in Jurkat cells (acute lymphoblastic leukemia cell line). These effects are caused by the increased expression of p73 and activation of the p73 pathway that causes also the down-regulation of the anti-apoptotic and epigenetic integrator UHRF1 [151]. 

### Aurora Kinase A Inhibitors.

Aurora kinase A inhibitors appear very promising among the new classes of compounds that are undergoing clinical or preclinical validation as anticancer drugs [177]. In particular, the MLN8054 inhibitor, currently in clinical trials, was shown to induce the expression of TA-p73β, thus promoting p73 transcriptional activity and p73-dependent apoptosis in p53-null cells [152]. Indeed, Aurora kinase A behaves as a negative regulator of the p73 pathway; its overexpression was shown to inhibit TA-p73-dependent transcription of p53/p73 downstream genes, while its knock-down by means of siRNA gave opposite results [152].

### Imatinib.

The small molecule Imatinib (also known as Glivec or STI571 [178]) is a selective tyrosine-kinase inhibitor successfully used for the treatment of chronic myelogenous leukemia (CML). In particular, Imatinib inhibits the enzymatic activity of the oncogenic chimera BCR-ABL generated by the reciprocal translocation of chromosomes 9 and 22, the most common alteration observed in CML [178-180]. By interfering with c-Abl activity, Imatinib can potentially affect p73 functions both in BCR-ABL expressing cells and in normal cells. In CML-derived K562 cells, treatment with Imatinib increased the levels of phosphorylated p38, Chk2 and TA-p73, promoted co-localization between TA-p73 and PML, and induced TA-p73- and p38-dependent apoptosis [181]. On the contrary, other reports confirmed that Imatinib can dampen the upstream signaling converging on p73 activation, thus suggesting its use as a tool to investigate the c-Abl TA-p73 relationship *in vitro* [174, 182], as well as a useful molecule to reduce or avoid improper TA-p73 activation and apoptosis *in vivo *[183].

### mTOR Inhibitors.

Finally, we would like to place particular attention on the recent discovery of a functional connection between the mTOR signaling and the p73 pathways [154, 155], which is particularly relevant given the high number of inhibitors available for mTOR and for its upstream regulators PI3K and AKT [184, 185]. The serine-threonine kinase mammalian target of rapamycin (mTOR) is deeply involved in the regulation of protein translation, cell growth, and metabolism and is deregulated in several pathologies, such as aging, metabolic diseases and cancer [155, 185-187]. Recently, an innovative approach based on analysis of gene expression signatures of cells treated with bioactive molecules suggested that mTOR inhibitors might be positive regulators of the p73 pathway. Indeed, RNAi mediated knockdown of mTOR, or treatment with various mTOR inhibitors (e.g. rapamycin [188] or metformin, see also Table **1** [188]), resulted in the specific accumulation of TA-p73β and in its transcriptional activation [154, 155]. The mechanism of TA-p73β stabilization after mTOR inhibition is still unknown, but seems to occur at the post-translational level.

These results point out the attractive possibility of stimulating p73 functions by using the already available plethora of inhibitors for mTOR or for its upstream regulators, administered alone or in combination with standard chemotherapy [184-186]. For instance, co-administration of rapamycin and cisplatin is effective in inducing p73-dependent cell death in breast cancer basal-like cells expressing mut-p53, representing a potential useful therapeutic strategy that is currently investigated in a clinical trial (NCT00930930) [156].

Also α-Tocopherol ether-linked acetic acid (α-TEA), a promising pro-apoptotic small bioactive lipid, can also evoke p73 activation/response by the inhibition of the mTOR pathway. Indeed, by promoting the JNK-mediated inhibition of IRS-1, an upstream regulator of the PI3K/AKT/mTOR pathway, α-TEA may be considered as an indirect potential activator of TA-p73 stabilization triggered by mTOR inhibition [159]. The combination of α-TEA with doxorubicin or cisplatin was investigated in basal-like triple-negative breast cancer, demonstrating that the two combinations act synergistically, enhancing apoptosis triggered by the activation of p73 transcriptional activity [160].

Finally, also the natural compound curcumin, a molecule that is currently employed in several clinical trials, could impair the mTOR/p73 axis due to its ability to disrupt the mTOR interaction with its partner Raptor, thus blocking mTOR-complex activity [158]. Interestingly, curcumin may potentially affect p73 functions also through the inhibition of NF-kB (and the subsequent stabilization of p73, see above), through the inhibition of IkB kinase and the subsequent phosphorylation of IkBα [189]. Accordingly, it was recently reported that curcumin treatment triggers p73 accumulation and p73-dependent apoptosis in several p53-null cancer cell lines, but not in normal cells [157]. 

## CONCLUSIONS

**Fig. (2):**
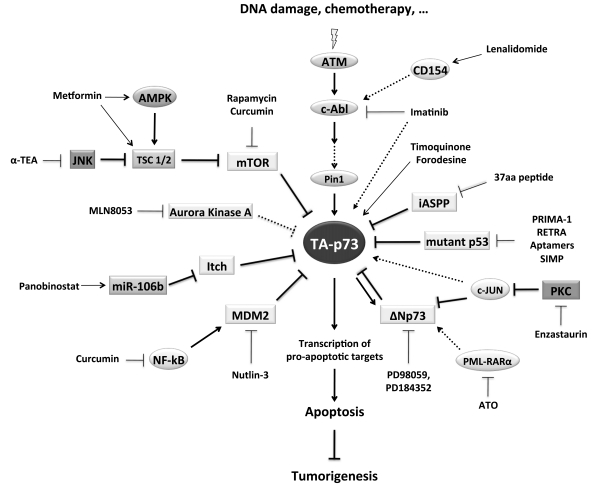
The p73 pathway and its regulators. A simplified view of the p73 pathway, of its regulators, and of the molecules that directly or indirectly can modulate its function. See text for details.

Solid experimental and clinical evidences indicate that TA-p73 isoforms can potentially vicariate p53 functions in cancer cells by inducing apoptosis after DNA damage. The p73 pathway is therefore an attractive target for cancer drug development. Considering that p73 is a transcription factor, it is difficult to imagine a molecule or compound that could directly act on it, but we have provided here several examples demonstrating the potential efficacy of pharmacological tools that can modulate levels and activities of p73 by acting on its upstream regulators (Table **1** and Fig. **2**). The evidences are more than sufficient to encourage further development of such tools and to justify investments for the identification of additional drugs (new or existing) that may target known important regulators of p73 (e.g. Itch inhibitors).

In parallel, we need to improve our knowledge of the molecular basis of p73 expression and regulation, aiming to a “global” or “system-level” comprehension of the signaling pathways that trigger tumor-suppressive activities of p73 in response to DNA damage and other stimuli. Such knowledge will be instrumental to reach the following crucial objectives. 1) To develop diagnostic markers of the “p73 pathway status” that could predict whether a p73-activating therapy would be appropriate in a given tumor and perhaps to direct the choice of which upstream p73 modulator should be targeted by a new generation of pathway-tailored drugs. In fact, the efficacy of any drug aimed at modulating the p73 pathway is going to be affected by cell-specific parameters: for example, which p73 isoforms are actually expressed and at what relative levels and what is the status of the upstream p73 regulators that are targeted by the drug under consideration. 2) To suggest the potential “repositioning” [190, 191] of drugs that are already in clinical use for other diseases, but can directly or indirectly affect molecular components of the p73 signaling pathway, as strikingly exemplified by the case of mTOR inhibitors described above. 3) To identify additional components of the p73 pathway that might in general influence the outcome of p73-targeted therapies, and may themselves become molecular targets for novel drugs, or repositioning of existing compounds.

We are confident that molecular insights derived from basic and clinical research integrated with next generation post-genomic tools and the use of gene expression signatures to discover connections among pathways and drugs will significantly increase – in the near future – the number of drugs potentially targeting the p73 pathway.

## Figures and Tables

**Table 1.** List of Drugs Targeting the p73 Pathway

Drug	Target	Mechanism of Action	Effects	Reference
RETRA	Mutant p53 (?)	Displacement of mut-p53/p73 complex	p73-dependent inhibition of cell growth *in vitro* and *in vivo* in tumor xenografts	[138]
Aptamers	Mutant p53	Unknown	Apoptosis in mut-p53 expressing cells	[139]
SIMP peptides	Mutant p53	Displacement of mut-p53/p73 complex	p73-dependent apoptosis in mut-p53 expressing cells in combination with chemotherapeutic drugs (doxorubicin)	[140]
Prima-1, Prima-1^MET^ (APR-246)	Mutant-p53	Reactivation of mutant p53 through covalent binding to their core domain	Apoptosis in tumor cells expressing mutant-p53, alone or in combination with cisplatin	[141, 142]
37aa peptides	iASPP	Displacement of iASPP-p73 complex	p73-dependent apoptosis *in vitro* and in tumor xenograft *in vivo*	[126]
Nutlin-3	MDM2	Displacement of MDM2-E2F1-p73 complex	Apoptosis in p53-null or mut-p53 expressing cells in combination with chemotherapeutic drugs (doxorubicin)	[143-145]
Forodesine	Unknown	Increased TA-p73 transcription	Apoptosis in CLL cells, alone or in combination with bendamustine and rituximab	[146]
Lenalidomide (CC-5013, or Revlimid)	Unknown	Induction of CD154 expression, that trigger the c-Abl-mediated activation of p73	CD95-mediated or ﬂudarabine-induced c-abl/p73 dependent apoptosis in p53-deficient CLL cells	[147]
Panobinostat (LBH589)	HDACs	E2F1- and myc-mediated transcription of miR-106b, that targets the p73 ubiquitin ligase Itch	TA-p73 induced apoptosis in CLL cells	[148]
Arsenic trioxide (ATO)	PML-RARa	Reduction of ∆N-p73 levels and increase of p300-mediated acetylation of p73 in APL cell lines. Increased expression of both TA- and ∆N-p73 expression in primary APL cells.	p73-mediated apoptosis alone, greatly increate by co-administration with MEK1 inhibitors (PD98059, PD184352)	[149]
PD98059, PD184352	MEK1	Alteration of TA/∆N-p73 ratio: reduction of ∆N-p73 levels and the accumulation and tyrosine phosphorylation of TA-p73	p73-mediated apoptosis alone in co-administration with ATO	[149, 150]
Thymoquinone	Unknown	Increased TA-p73 protein level	p73-dependent cell cycle arrest and apoptosis in acute lymphoblastic leukemia (ALL) Jurkat cell line	[151]
MLN8054	Aurora kinase A	Induction of TA-p73β expression	p73-dependent apoptosis in p53-null cells	[152]
Enzastaurin (LY317615.HCL)	PKC kinases	Accumulation of β-catenin, that promotes c-Jun-dependent induction of p73	p73-dependent apoptosis in multiple myeloma cells	[153]
Rapamycin	FKB12	Direct mTOR inhibition and increased TA-p73β levels	p73-dependent cell death, increased by cisplatin co-administration in basal-like triple negative breast cancer cells	[154-156]
Metformin	AMPK	Increased inhibitory Ser789 phosphorylation of IRS-1, decreased PI3K/AKT activation, increased phosphorylation of TsC2, inhibition of Rheb, inhibition of mTOR, increased TA-p73β levels	Not investigated	[155]
Curcumin	mTOR, NF-kB	NF-kB and mTOR inhibition and TA-p73 accumulation and activation	p73-dependent apoptosis	[157, 158]
α-TEA	Unknown	Increased p73 transcriptional activity, downstream of c-Abl, JNK and Yap. Indirect JNK-mediated inhibition of mTOR, increased TA-p73 levels	p73-dependent apoptosis in basal-like triple negative breast cancer cells	[159, 160]
